# Functionalization of Se-Te Nanorods with Au Nanoparticles for Enhanced Anti-Bacterial and Anti-Cancer Activities

**DOI:** 10.3390/ma15144813

**Published:** 2022-07-10

**Authors:** Shahin Shah Khan, Irfan Ullah, Shah Zada, Aftab Ahmad, Waqar Ahmad, Haijun Xu, Sadeeq Ullah, Luo Liu

**Affiliations:** 1 College of Life Science and Technology, Beijing University of Chemical Technology, Beijing 100029, China; khan@mail.buct.edu.cn (S.S.K.); irfan@mail.buct.edu.cn (I.U.); aftabbiochem@yahoo.com (A.A.); waqar@mail.buct.edu.cn (W.A.); hjxu@mail.buct.edu.cn (H.X.); 2 Beijing Key Laboratory for Bioengineering and Sensing Technology, Research Center for Bioengineering and Sensing Technology, School of Chemistry and Biological Engineering, University of Science and Technology Beijing, Beijing 100083, China; shahzadabuneri10@gmail.com

**Keywords:** near infrared, anti-biofilm, *Escherichia coli*, *Staphylococcus aureus*, Se-Te, Se-Te@Au, anti-cancer activity

## Abstract

The use of medical devices for therapeutic and diagnostic purpose is globally increasing; however, bacterial colonization on therapeutic devices can occur, causing severe infections in the human body. It has become an issue for public health. It is necessary to develop a nanomaterial based on photothermal treatment to kill toxic bacterial strains. Appropriately, high photothermal conversion and low-cost powerful photothermal agents have been investigated. Recently, gold nanocomposites have attracted great interest in biological applications. Here, we prepared rod-shaped Se-Te@Au nanocomposites of about 200 nm with uniform shape and surface-coated with gold nanoparticles for the first time showing high anti-bacterial and anti-cancer activities. Se-Te@Au showed proper structural consistency and natural resistance to bacterial and cancer cells. The strong absorption and high photothermal conversion efficacy made it a good photothermal agent material for the photothermal treatment of bacterial and cancer cells. The Se-Te@Au rod showed excellent anti-bacterial efficacy against Gram-negative *Escherichia coli* and Gram-positive *Staphylococcus aureus,* with highest recorded inhibition zones of 25 ± 2 mm and 22 ± 2 mm, respectively. More than 99% of both types of strains were killed after 5 min with a near-infrared (NIR) laser at the very low concentration of 48 µg/mL. The Se-Te@Au rod’s explosion in HeLa cells was extensively repressed and demonstrated high toxicity at 100 µg/mL for 5 min when subjected to an NIR laser. As a result of its high photothermal characteristics, the exceptional anti-bacterial and anti-cancer effects of the Se-Te@Au rod are considerably better than those of other methods previously published in articles. This study could open a new framework for sterilization applications on the industrial level.

## 1. Introduction

The use of medical devices for therapeutic and diagnostic practices has many consequences, the risks of which are not negligible, specifically when such devices are planned to be implanted inside the body for a long time. The most important related problem is bacterial colonization on therapeutic devices after surgeries, which has a significant impact on the health of the patient. The treatment of infectious diseases is associated with a certain cost for the patients [1]. These obstacles are usually associated with bacteria causing the contamination of surgical wounds, resulting in nosocomial infection [1]. Approximately 20 lakhs of nosocomial infections occur in the United States of America that cost nearly USD 11 billion annually [2]. These infections are extremely difficult to handle because of biofilm-forming bacteria (e.g., MRSA (methicillin-resistant *S. aureus*) and VRE (vancomycin-resistant Enterococcus)), which are highly resistant to conventional antibiotic therapies and are very dangerous for patients’ health [3,4]. Recently, a study was published by European Antimicrobial Resistance Surveillance Network (EARS-Net). They demonstrated that in 2015, more than 30,000 deaths occurred in Europe due to antibiotic-resistant bacterial infections [5]; the same report was recently published in 2022 and demonstrated that each year, more than 670,000 infections are due to bacteria resistant to antibiotics and that approximately 33,000 people die as a direct consequence [6].

In recent times, a wide range of bactericidal agents have been reported, such as microbicidal peptides [7,8] polymers [9,10], quaternary ammonium salts [11], and inorganic nanoparticles (NPs) [12,13,14,15,16]. Amongst them, inorganic NPs that include gold (Au) [17,18], silver (Ag) [13,19], copper (Cu) [20,21], and other metals [22,23] have brought new opportunities for developing effective and safer bactericidal therapeutics. Especially, AgNPs have become the agents that are most commonly applied for the treatment of burns, wounds, and many other infectious diseases because of their excellent bactericidal properties against a wide range of microbes, including fungi, virus, pathogenic bacteria, and many other eukaryotic organisms [22,24].

Metallic nanostructures can interact with the electromagnetic radiations from their electrons, producing quantum effects [25]. One of these effects is the phenomenon of plasmon resonance, in which incident light striking metal nanostructures oscillates the electrons at a resonant frequency in these NPs [26]. In addition, plasmonic NPs act as sensors by transducing the bands of absorption in their UV spectra [27]. Au-P-Au-coupled AuNPs were developed to promote significant surface-enhanced Raman scattering (SERS) results, obtaining a maximum enhancement factor of 1.36 × 10^8^ for detection of glucose. The united nanofabrication technology for Au-P-Au-coupled Au NP platforms with reasonable SERS enhancement encourages applications in nonlinear optics, nano-photonics, and surface-enhanced spectroscopy. Typical plasmonic nanocomposites with strongly enhanced electric fields normally possess at least one of the following features: nanogaps, nanotips, or nanocavities [28]. In another study, a localized surface plasmon resonance (LSPR)-based sensor using a narrowband metamaterial absorber based on an Au nanocomposite array was examined. The device showed extensive performance, such as good absorption, polarization insensitivity, perfect sensitivity, and FOM, making it appropriate for practical uses [29]. A bio-inspired material was constructed for the plasmonic detection of ACE through AuNPs [30]. AuNP dimers and MXenes showed good SERS enhancement and reproducibility. The constructed SERS aptasensor allowed aflatoxin B1 (AFB1) to be detected over a wide linear range. The novel AuNP dimers/MXenes are composites constituted as an effective SERS platforms [31]. Noble-metal NPs such as AuNPs and AgNPs can produce ultrasensitive SERS signals owing to their plasmonic properties. AuNPs have been widely studied for their biocompatibility and potential to be used in clinical diagnostics and therapeutics or combined for theranostics. The results showed that when using 785 nm laser excitation, the SERS signal increased with the increase in the size of AuNPs up to 60 or 80 nm [32]. A dual-mode electrochemical biosensor was successfully developed for the simultaneous detection of two different kinds of breast cancer biomarkers, namely, cancer antigen 15-3 (CA 15-3) and microRNA-21 (miRNA-21), for the first time. The sensor was based on a two-screen-printed carbon electrode array (dual electrode) modified by poly(3-aminobenzylamine)/two-dimensional (2D) molybdenum selenide/graphene oxide nanocomposites and individually functionalized with 2,3-diaminophenazine–gold NPs and toluidine-blue–gold NPs [33]. A rapid and colorimetric nano-biosensor was constructed employing AuNPs to target platelet-derived growth factor (PDGF), a circulating biomarker that is up-regulated in plasma in prevalent ovarian cancer. The results presented in a research study which can imply in practical application of aptamers and AuNPs for cancer diagnosis, exhibiting their benefits of reliability, selectivity, and reproducibility [34]. The intensive near-infrared radiation (NIR) absorption of NPs causes the photothermal heating of their surroundings, generating radicals used for therapeutic applications [35,36,37]. Many of the inorganic NPs have been evaluated on different implantable devices for their potential application as antimicrobial agents [38]. For example, silver NPs were used to functionalize catheters, as they released Ag^+^ ions upon oxidation, which could disrupt the membrane of bacteria by binding to DNA, and inhibit the synthesis of proteins [39]. There is an increasing use of AuNPs in the field of biomedical science [40,41,42]. Particularly, the interaction of GNPs with light is very efficient in terms of localized surface plasmon resonance (LSP) [43]. While some part of the light that interacts is scattered elastically, the metal absorbs the remaining part, and at the end, it dissipates it in the form of heat to its surroundings [44,45]. GNPs’ capability of delivering heat when locally illuminated was thoroughly investigated, and it was used for therapies based on hyperthermia, i.e., cancer and other diseases [46]. Furthermore, the use of plasmon-enabled site-directed hyperthermia on surgical implants in the context of disinfection to eliminate bacterial contamination was also investigated [47]. Similarly, up to 80% of the infections of *Pseudomonas aeruginosa* and *Chromobacterium violaceum*, which create a biofilm on the wound due to antibiotic resistance, were inhibited by SeNPs and TeNPs [48]. Along the same line, an in vitro collagen-matrix wound model was used to eradicate multidrug-resistant *Acinetobacter baumannii* using selenium [49]. Against both Gram-negative and Gram-positive bacteria, TeNPs showed an important anti-bacterial activity in a concentration range from 5 to 50 μg mL^−1^ over 24 h [50]. In addition, Se nanorods and Se-Au composites that damage the cytoplasmic membrane were used against *S. aureus* and *E. coli* [51].

In the current study, an NIR-light-activated photothermal therapeutic strategy was developed; it was not only able to eliminate the Gram-positive and Gram-negative strains of *S. aureus* and *E. coli*, respectively, but could also inhibit cancerous cells thanks to its synergistic effect. It was assumed that bacterial membrane disruption could be boosted by the synergistic effect and this could also accelerate the release of both Selenium (Se)-loaded tellurium (Te) ions and selenium–tellurium-loaded gold (Au) ions with the stimulation of NIR-laser irradiation. Our prepared materials showed good anti-bacterial activity in a smaller amount of time against both positive and negative bacterial strains. It had better results than positive antibiotic tobramycin, as used in this study. As compared with other nanoparticles, up to 200 micrograms of NPs exhibited good invitro anti-cancer activity. Furthermore, it also had very good activity without NIR irradiation.

## 2. Experimental Section

### 2.1. Strains and Chemicals

The chemicals used in this study were purchased from Sigma-Aldrich, St. Louis, MO, USA, i.e., sodium selenite (Na_2_SeO_3_) (purity of 99%), telluric acid (H_6_TeO_6_) (99%), gold salts (99.99%), hydrazine (98%), ascorbic acid (99%), cetyltrimethylammonium bromide (CTAB) (99%), 2,2,6,6-tetramethylpiperidine (99%), 5,5-dimethyl-1pyrroline-N-oxide (97%), 4′,6-diamidino-2-phenylindole (DAPI) (98%), and Sytox TM Green dead cell stain (dye) (Thermofisher). Bacterial strains *Escherichia coli* (ATCC8739) and staphylococcus aureus (ATCC 6538) were provided by China General Microbiological Culture Collection Center (Chinese Academy of Sciences).

### 2.2. Synthesis of Se-Te Alloy Nanorods

Se-Te alloy nanorods were prepared by mixing two reducing agents, i.e., ascorbic acid and hydrazine; the reduction of both salts was conducted in micellar solution. Briefly, both salts, H_6_TeO_6_ (20 mM) and Na_2_SeO_3_ (20 mM), were separately prepared in the presence of CTAB (2 mg/mL), and the final volume of the solution was raised to 100 mL. Afterward, it was sonicated for 15 min, followed by constant stirring (250 rpm) in an oil bath for 3 h at 95 °C. Then, a mixture of reducing agents (ascorbic acid (1000 mg) and hydrazine (500 µL)) was prepared in 10 mL, slowly added to the reaction above, and maintained for 30 min at 95 °C. Finally, a color change abruptly appeared from colorless to deep gray. The product was collected and purified using centrifugation; then, it was dried overnight at 60 °C [52].

### 2.3. Synthesis of Se-Te@Au Alloy Nanorods 

The CTAB-mediated positively surface charged Se-Te alloy nanorods were dispersed into double deionized water (300 mg of particles in 30 mL) with 30 min of sonication assistance. After that, gold salts (2 mM in 10 mL solution) were sonicated for 30 min and added drop-wise to the synthesized Se-Te rod solution. The mixture was vigorously stirred for 2 h at 60 °C; then, 3 mL of hydrazine was added to reduce gold salts to enhance loading on Se-Te alloy nanorods. Finally, the Se-Te@Au nanomaterial was obtained by centrifugation and dried at 60 °C overnight.

### 2.4. Nanoparticle Characterization

The crystallinity, binding energy, and formation of the synthesized NPs were confirmed by using different analytical techniques. The morphologies of the synthesized nanomaterials were measured by transmission electron microscopy (TEM; Phillip CM12, Phillip, Amsterdam, The Netherlands) and scanning electron microscopy (SEM; Hitachi, Tokyo, Japan, SU 8010 Japan) using the energy-dispersive X-ray method (EDX; Phillips XL30 ESEM) for elemental composition. The composition of bimetallic nanoalloys Se-Te@Au and Se-Te with individual elements’ binding energies were studied using XPS (Scienta, Uppsala, Sweden; R 3000 pass energy, 50 eV angular mode).

For singlet-oxygen and hydroxyl-radical generation, ESR was performed using a JEOL JES FA 100 instrument, JES, Wanchai, Hong Kong. Treatment was performed using an NIR laser operating at 808 nm (Infrared Diode Laser system, Changchun CNI China, Changchun, China). The temperature change that was mediated by Se-Te@Au and Se-Te was tested at different power densities using an NIR laser operated at 808 nm.

### 2.5. In Vitro Anti-Bacterial Activity

The anti-bacterial activities of Se-Te and Se-Te@Au were assessed using Gram-positive *S. aureus* and Gram-negative *E. coli* bacterial strains. Lysogeny broth medium (LB) was used containing bacterial cells, and different concentrations (6, 12, 24, and 48 μg/mL) of Se-Te and Se-Te@Au were added. The illumination of the solution was performed for 10 min (1.00 W/cm^−2^) without/with an 808 nm laser [37,53]. Then, after incubating for 24 h, the solution was shifted to 96-well plates. Afterwards, the optical density (OD) was recorded at an absorbance of 600 nm at an interval of one hour for ten hours to calculate the concentration of bacteria using a multimode microplate reader (Spectra Max M5). This was followed by 10^6^-fold dilution of the bacterial suspension, of which only 100 microliters were transferred to LB plates and grown for 24 h at 37 °C. Then, the inhibition zones were recorded for each concentration under dark and light conditions.

### 2.6. Morphological Characterization of Bacteria

The fluorescent-based dead cell method was used for the bacterial death assay. A volume of 1500 μL of bacterial cells was collected and rinsed with phosphate-buffered saline (PBS; pH 7.4); subsequently, it was treated with a 48 μg/mL concentration of Se-Te@Au and Se-Te and irradiated with NIR light at an absorbance of 808 nm and a power of 1.00 W/cm^−2^ for 10 min. Fluorescent dye propidium iodide (30 μM, 50 μL) was introduced after culturing the mixture for one hour, and it was then incubated for 15 min. For the observation of the samples, an inverted fluorescence microscope (Leica DMI; 4000B; Danaher, Duesseldorf, Germany) was used.

The bacterial cells were harvested, rinsed three times, and grown with a concentration of 48 μg/mL of Se-Te@Au and Se-Te for one hour for SEM analyses. After NIR-laser exposure for ten minutes, all the collected samples were centrifuged at 5000 rpm and then washed with PBS. Afterwards, using glutaraldehyde 2.5 percent solution, the cells were fixed on a glass and then washed with PBS, ethanol-dehydrated, and vacuum-dried. They were imaged using a scanning electron microscope (SEM; Hitachi SU, 8080 Japan, Hitachi, Tokyo, Japan).

## 3. Result and Discussion

Recently, the researcher had reached to a consensus on the biological properties of selenium [54,55], while the biological activity of tellurium has rarely been reported, and research has been limited to its electrochemical and optical properties. Therefore, in the current study, selenium-loaded tellurium and selenium–tellurium-loaded gold nanoparticles were obtained, and an NIR-light-activated photothermal therapeutic strategy was followed; this was not only able to eradicate Gram-positive and Gram-negative strains *S. aureus* and *E. coli*, respectively, but could also inhibit cancerous cells due to having a synergistic effect [56]. The disruption of the bacterial membrane could be boosted due to the synergistic effect, and the release of selenium (Se)-loaded tellurium (Te) ions and selenium–tellurium-loaded Gold (Au) ions could also be increased with the stimulation of NIR irradiation. As compared with other NPs, up to 200 micrograms of nanoparticles exhibited good invitro anti-cancer activity. Furthermore, it also had very good activity without NIR irradiation.

### 3.1. Characterization

The Se-Te and Se-Te@Au morphological forms were studied by SEM (scanning electron microscopy) and TEM (transmission electron microscopy) operating at 15.0 and 200 kV, respectively. HRTEM was used to obtain the spectral and elemental mapping of the synthesized nanomaterials, the Se-Te and Se-Te@Au rods. The binding energies of Au, Te, and Se in the synthesized nanomaterial composites were studied using XPS.

#### 3.1.1. Electron Microscopy of Se-Te Nanorods 

The nanorods were synthesized and characterized using different analytical techniques (SEM, TEM, EDX, XPS, and XRD), as previously published [52].

#### 3.1.2. Scanning Electron Microscopy of Se-Te@Au Nanorods 

**Figure 1:**
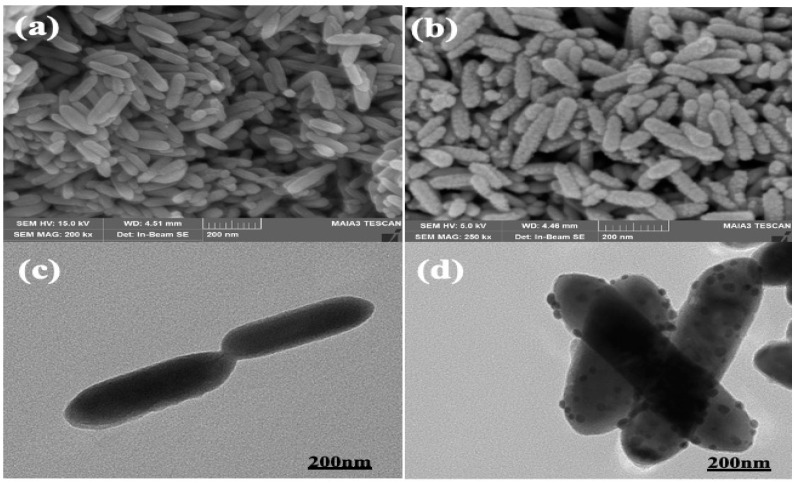
SEM micrographs of Se-Te and Se-Te@Au nanorods, respectively (**a**,**b**). TEM images of Se-Te and Se-Te@Au rods, respectively (**c**,**d**).

**Figure 2:**
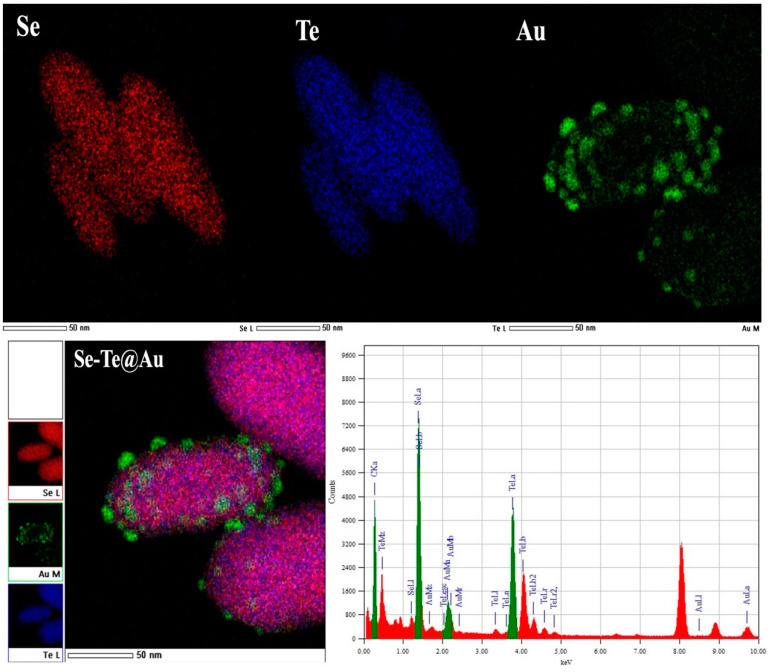
EDX spectral profile and HR-EDX mapping of the synthesized Se-Te@Au nanorods.

Gold-loaded Se-Te nanorods of uniform length and shape were synthesized at 95 °C, and their morphologies were studied by SEM and TEM, respectively. The nanorods synthesized at the optimized temperature of 95 °C had a uniform size of about 200 nm and were about 60–70 nm in diameter. Moreover, the synthesis of the nanorods was affected by the reducing agent used. Ascorbic acid combined with hydrazine resulted in well-distributed and monodispersed nanorods, as shown in Figure 1a. Afterward, gold NPs were dispersed on the surface of nanorods as shown in Figure 1b, where the gold NPs can be clearly seen on the surface of nanorods; they converted the smooth surface into a rough surface, although there were no significant changes in the diameter or in the size of nanorods. The photographs obtained by SEM (Figure 1a,b) analysis were further confirmed by TEM as shown in Figure 1c,d. From these figures, it was further confirmed that the AuNPs on the surface of Se-Te nanorods were successfully dispersed with strong attachment. The metalloid and Se-Te@Au nanorods certified the higher active mass utilization during electrochemical reactions [52]. The nanorods’ basic spectral outline obtained by EDX showed the amalgamation of the two metalloids, which was confirmed with high-resolution mapping. Furthermore, the spectra obtained from EDX showed all the elements, Te and Se, and the presence of supported AuNPs. The formation of the alloy nanorods was also verified by HRM-EDX mapping. All the elements were represented by different colors; AuNPs were clearly observed on the outer surface of Se-Te nanorods, as shown in Figure 2.

#### 3.1.3. XPS Spectral Analysis

**Figure 3:**
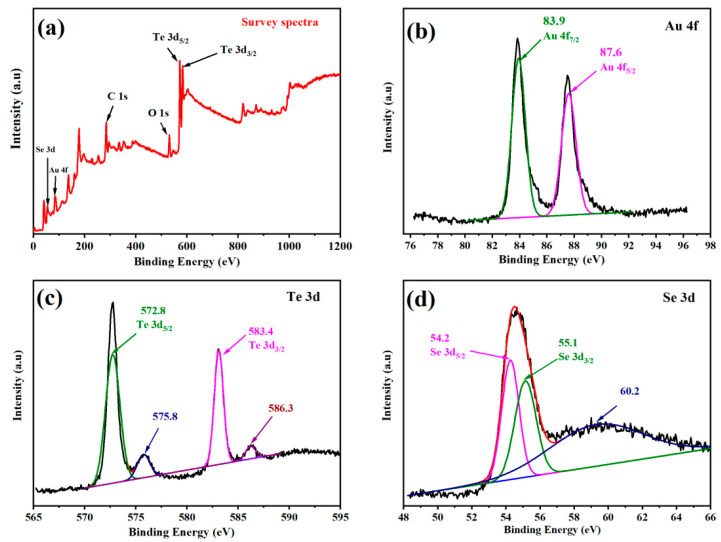
XPS spectral patterns of the Se-Te@Au composite showing full (**a**) and deconvoluted spectra of (**b**) Au 4f, (**c**) Te 3d, and (**d**) Se 3d, respectively.

X-ray photoelectron spectroscopy (XPS) analysis was used to study the composition of Se-Te@Au nanocomposite materials and the binding energies of each element in the composite materials, and the obtained spectra are shown in Figure 3a. The composite nanorods’ full survey spectra confirmed the individual elements of Te, Se, O, and Au, as shown in Figure 3. In a similar way, the individual elements’ XPS spectra were further deconvoluted, and the obtained results can be observed in Figure 3. Two major peaks could be observed on the binding energies: 83.9 indicates Au 4f_7/2_ and 87.6 eV Au indicates 4f_5/2_ in Figure 3b; this confirmed the presence of AuNPs on the bimetallic Se-Te nanorods. Four different spectral bands were shown by Te 3d deconvolution spectra at binding energies of 572.8, 575.8, 583.4, and 586.3 eV. The peaks obtained at 572.8 eV and 583.4 eV corresponded to 3d_5/2_ and 3d_3/2_ of Te (0) 3d, while the two other bands were attributed to Te (IV) 3d, as shown in Figure 3c. The coexistence of the two forms indicated that Te existed in both the oxide and elemental forms [57]. Furthermore, Figure 3d indicates the spectrum of Se 3d, revealing two different bands at 54.2 eV (Se 3d_5/2_) and 55.1 eV (Se 3d_3/2_), while around 60.2 eV a broad band was observed which was assigned to the SeO_2_ bond in the Se-Te@Au composite. The results confirm that our desired material (Se-Te@Au) was successfully prepared.

### 3.2. Anti-Bacterial Activity

The biosynthesized Se-Te and Se-Te@Au NPs’ anti-bacterial efficacies were investigated under light and dark conditions against the commonly existing pathogenic bacterial strains *S. aureus* and *E. coli*. Se-Te and Se-Te@Au shows improved activity levels and were exceptionally active against *E. coli*, and zones of inhibition of 24 ± 2 mm and 25 ± 2 mm were recorded, respectively. They also exhibited significant activity against *S. aureus*, having inhibition zones of about 21 ± 2 mm and 22 ± 2 mm, respectively. The exceptional activity against *E. coli* may be due the structural differences in the cell wall composition. The cell wall of *S. aureus,* Gram-positive bacteria, is composed of a thick peptidoglycan layer, which is thinner in Gram-negative *E. coli*. Less hindrance should be provided for particles to enter through the thin peptidoglycan layer of *E. coli*. Different mechanisms are used to eliminate bacteria from noble metals using NPs, including damaging their membrane, ROS generation, damaging their DNA, and inhibiting some of the vital enzymes [13,58,59]. Among these mechanisms, ROS generation is a principal mechanism that kills the microorganisms by damaging their cell membrane, nucleic acids, and proteins [60,61]. The current results in comparison with previously reported data are highlighted in Table 1.

**Figure 4:**
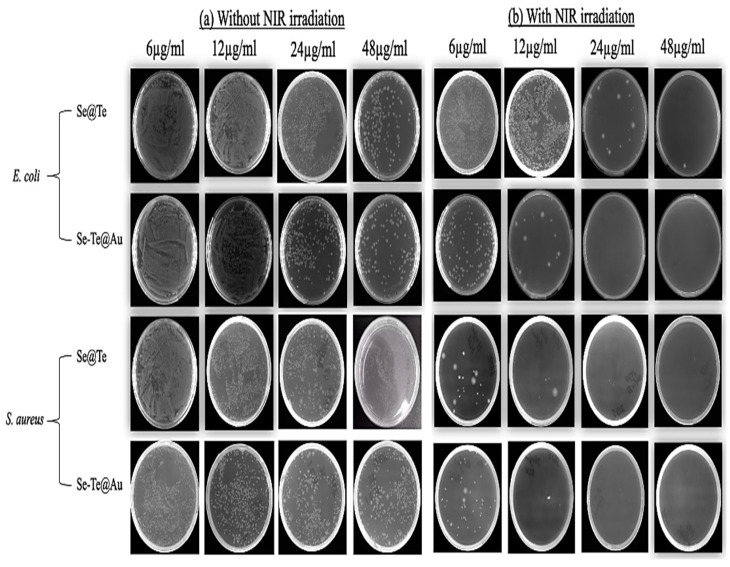
Antibacterial activity (**a**) shows the activity of Se-Te and Se-Te@Au treated without NIR against *E. coli* and *S. aureus* with different concentration. (**b**) Represents the activity of Se-Te and Se-Te@Au with NIR in different concentrations.

Furthermore, the anti-bacterial activities of Se-Te and Se-Te@Au NPs were observed at the different concentrations of 6 µg, 12 µg, 24 µg, and 48 µg with NIR light and without NIR light, with promising results being observed against *S. aureus* and *E. coli* for both NPs (Figure 4b) with NIR light. On the other hand, without NIR light, excellent results were observed using 48µg of these NPs against *E. coli* and *S. aureus*, as shown in Figure 4a.

#### 3.2.1. Growth Curves of Microbial Cells Treated with Different Concentrations of NPs

**Figure 5:**
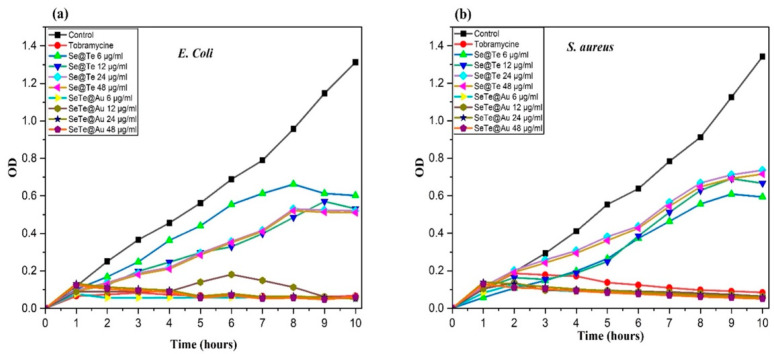
Growth curves of bacterial cells (**a**) *E. coli* (**b**) *S. aureus* exposed to different concentrations of Se-Te and Se-Te@Au.

The growth curves of bacteria showed in Figure 5 demonstrate the bacterial inhibition for all the tested concentrations of Se-Te and Se-Te@Au (6–48 μg/mL). Without the addition of these NPs, the culture media did not show any inhibition, and after 24 h, they reached their stationary phase. On the other hand, complete inhibition was observed at 12, 24, and 48 μg/mL of Se-Te and Se-Te@Au for Gram-negative (*E. coli*) and Gram-positive bacteria (*S. aureus*). However, at a concentration of 6 μg/mL, the bacterial growth was slightly inhibited but was not enough to outpace bacterial reproduction. The results obtained show that the anti-bacterial activity of NPs increased with the increase in concentration. Faster growth inhibition was exhibited by Se-Te@Au in both *S. aureus* and *E. coli*. Growth inhibition was maximum for *E. coli* at concentrations of 24 and 48 μg/mL.

#### 3.2.2. Live–Dead Assay

**Figure 6:**
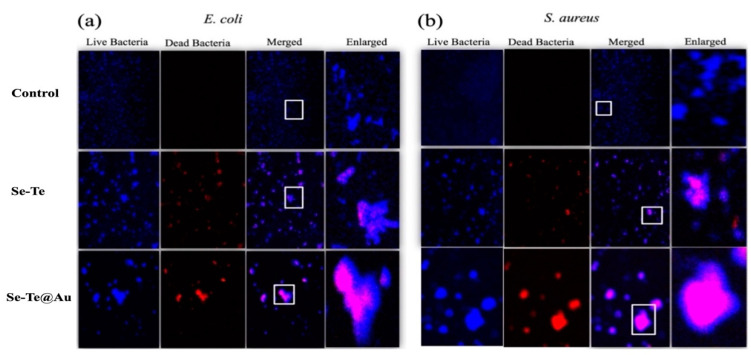
(**a**) Representative fluorescence images of *E. coli* cells after 2 h of treatment. The dead cells were visualized by PI staining (red), while DAPI (blue) helped to identify live cells. Scale bar is 25 μm. (**b**) Fluorescence images of *S. aureus* cells after 2 h of treatment. The dead cells were visualized by PI staining (red), while DAPI (blue) showed live cells.

The determination of membrane damage was performed using a DNA-binding fluorescent dye (propidium iodide (PI)). PI dye was not absorbed by cells that had an intact membrane. Therefore, it could be suggested that PI bounded with DNA, shows membrane damage. Additionally, DAPI, a cell permanent nuclear staining agent was also used for staining the population of live bacteria as a normalizing factor (Figure 6a,b). The degree of membrane damage was determined from these two strains for each treatment group. No bacterial membrane damage was observed in the control group, while bacterial membrane damage was observed in groups treated with Se-Te and Se-Te@Au. The experiment was performed following a previously reported protocol [63]. The enhanced bacterial membrane damage by Se-Te@Au supports that the synergistic effect could be arise due to the hybrid design, showing highly efficient performance in destroying bacteria, and the need to homogeneously conjugate both active species into a single entity [37]. The enhancement of the damage to the membrane could be attributed to the increased interaction between the bacterial membrane and Se-Te@Au.

#### 3.2.3. Membrane Disruption

The disruption of the membrane caused by Se-Te and Se-Te@Au was confirmed with a PI staining assay (Figure 6). The Se-Te- and Se-Te@Au-treated bacterial cells were washed and then were treated with PI for 30 min. Normal cells did not absorb PI due to having an intact membrane, while PI was absorbed by cells having cell membrane damage [64]. Excited PI at 535 nm gave red fluorescence, while DAPI was used for the confirmation of the live bacterial cells and gave blue fluorescence. The treated cells’ extracellular morphologies exhibited a leakage of the cytoplasmic materials and cell membrane disruption in both strains at 48 µg/mL Se-Te and Se-Te@Au, respectively. In comparison to the control group, cellular deformation was more severe in the case of *E. coli* than in *S. aureus* [64,65].

**Figure 7:**
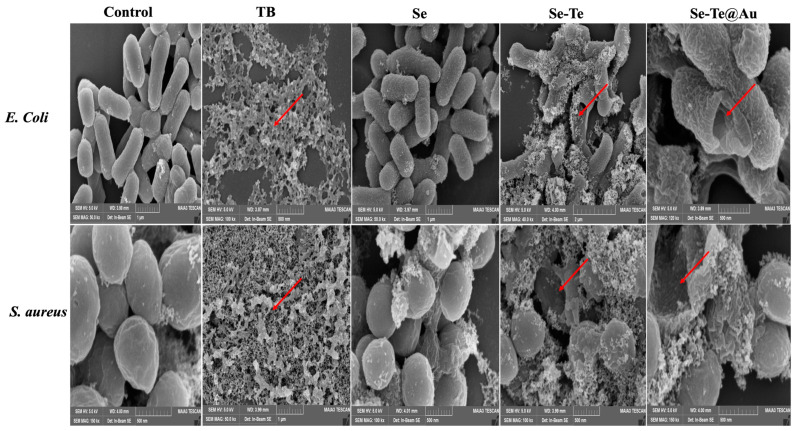
SEM images of *E. coli* control, TB, and *S. aureus* treated with Se, Se-Te, and Se-Te@Au nanoparticles; red arrows point towards cell leakage and severe disruption of cell membrane.

The current study reveals that Se-Te and Se-Te@Au-treated bacterial cells exhibited intracellular fluorescence, which confirmed the membrane damage of bacteria and the death of the cells. These results are in accordance with studies previously reported in the literature [61,62]. The treated cells’ extracellular morphologies showed the formation of pores and, subsequently, cytoplasmic materials leakage, which resulted in a deformed structure of bacterial cells in comparison with control cells (Figure 7).

#### 3.2.4. Photo-Thermal Efficacy of Se-Te and Se-Te@Au Nanorods

**Figure 8:**
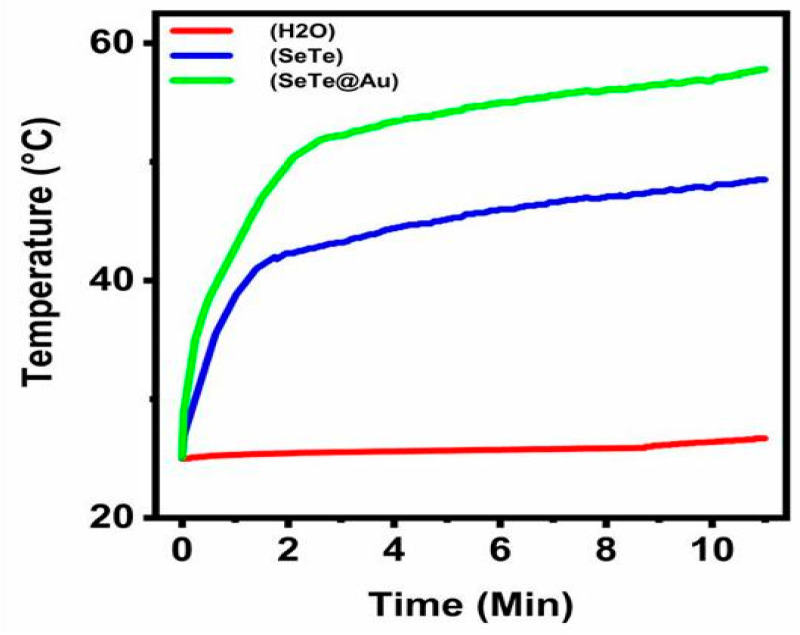
Temperature change measurement of Se-Te and SeTe@Au at a power of 1.00 W cm^−2^.

A comparison with the control was conducted to find out the photothermal potential of the designed NPs (Se-Te and Se-Te@Au). As depicted in Figure 8, the range of the temperature of the Se-Te and Se-Te@Au samples increased rapidly compared with the control sample during irradiation. For instance, the conversion of 1 mL (Se-Te@Au, 150 ppm) was reached at 58 °C within 10 min by applying NIR-laser irradiation. The rate of the photothermal conversion of the designed NPs (Se-Te and Se-Te@Au) was calculated by a method reported by Roper et al. with slight modifications [66]. These results indicate that Se-Te and Se-Te@Au NPs might be efficiently accelerated by NIR irradiation into heat.

#### 3.2.5. Investigation of ROS Production

**Figure 9:**
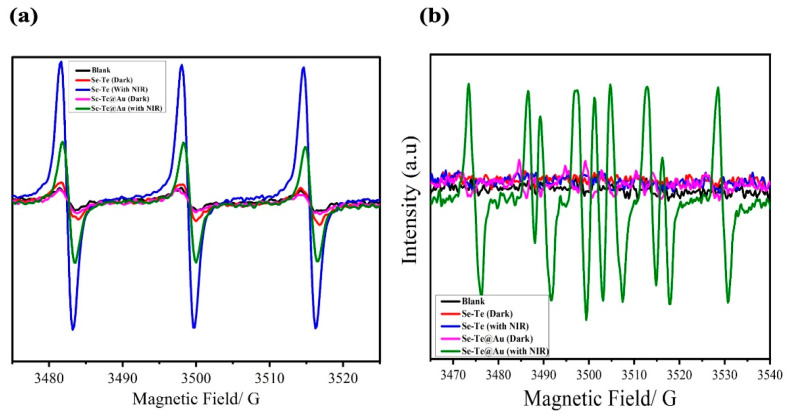
Electron spin resonance analysis. Spectra of single oxygen of control sample (**a**): Se-Te without NIR irradiation (red line) and irradiated with NIR light (blue line), Se-Te@Au without NIR (pink line) and with NIR (green line), and singlet oxygen species in the dark and exposed to visible light; (**b**) hydroxyl radical generation from the prepared samples.

Spectroscopic analyses were used to determine the mechanism of anti-bacterial activity of the reactive oxygen species (ROS), for example, by electron spin resonance (ESR), for finding the ROS types that promoted Se-Te and Se-Te@Au samples’ activities. Different conditions were used for ESR, i.e., in the presence of light and in the dark, along with irradiation by NIR light of (1.00 W cm^−2^, from 1–10 min). For the designed nanomaterials (Se-Te and Se-Te@Au), a trapping agent, TEMP (tetraethylemethyleproline), for the detection of singlet oxygen was used. The TEMPO adduct was produced by TEMP’s reaction with ^−1^O_2_. The singlet-oxygen induction signals were surpassed by Se-Te and Se-Te@Au in the spectra obtained by ESR both in the dark and under NIR-light irradiation, as shown in Figure 9. The lower-intensity signals of singlet-oxygen production may have been due to oxygen vacancy in the gold salts, as similar results have previously been reported [65]. It can be summarize that superoxide radical production occurs by atmospheric oxygen reaction with an electron from the surface of the desired hybrid nanomaterial, which is followed by the reaction with water molecules to produce hydrogen superoxide radicals, this is a phenomenon similar to that previously reported for other nanoparticles [17,67]. Due to recombination, the produced hydroperoxyl radicals yield H_2_O_2_ that could react with superoxide ions, producing hydroxyl ions [59]. Furthermore, a trapping agent for hydroxyl radicals, dimethylprolineoxide (DMPO), was used with the hybrid materials (Se-Te and Se-Te@Au). Under dark conditions, a lower-intensity signal peak was exhibited by the designed nanomaterials (Se-Te and Se-Te@Au), while after bombarding for 10 min with NIR light, prominent hydroxyl signals appeared in the tested samples. The successful generation of hydroxyl ions beside singlet oxygen by Se-Te and Se-Te@Au was observed in the presence of NIR light; the treated samples might be endorsed by proteins that are surface-capped and open oxygen vacancies in the NIR region. In NIR-light-treated samples, the excitation of the electrons could accelerate the soluble oxygen for the production of ROS. The literature shows that NPs, which present incorporated biologically capped moieties such as β hydroxypropyl-cyclodextrin, could synergistically enhance the generation of ROS in comparison with free NPs [61,68,69]. Upon radiation, our desire materials Se-Te@Au nanocomposites, produced ROS due to synergistic effect of bimetallic nanorods and gold nanoparticles. So, to summarize the discussion, singlet oxygen and hydroxyl ions were produced in the system due NIR radiation and are responsible for anti-bacterial and invitro anti-cancer activities.

### 3.3. Anti-Cancer Activity

**Figure 10:**
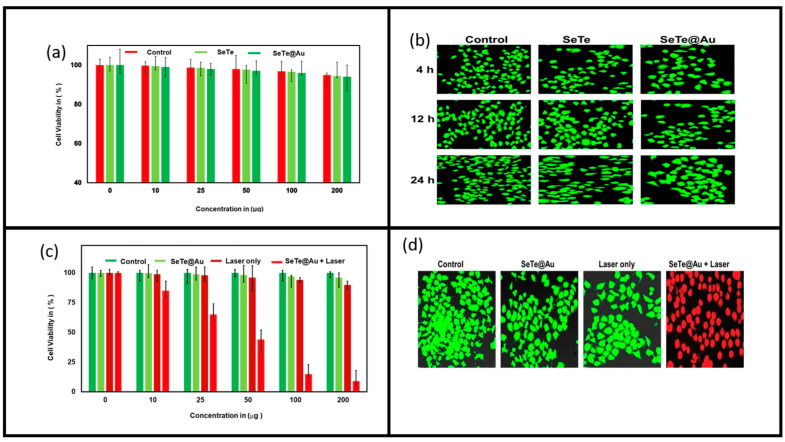
Cytotoxicity analyses of Se-Te and Se-Te@Au nanorods on HeLa cells using different concentrations (10, 25, 50, 100, and 200 µg/mL) (**a**), and their mapping analysis show in (**b**), at different interval of time (4, 12, and 24 h) the control, Se-Te@Au + laser with different concentration show in (**c**), and mapping analysis after 24 h are demonstrated in (**d**).

For the anti-cancer study, HeLa cells were treated with different concentrations of Se-Te and Se-Te@Au (10, 25, 100, and 200 µg) (Figure 10). A higher concentration of 200 µg Se-Te@Au did not show any toxicity, as demonstrated in the figure. Similarly, the same results were observed at different intervals of time (4, 12, and 24 h) in the control and Se-Te samples (Figure 10a,b). Cell viability was affected with Se-Te@Au + laser, which shows that the laser could activate the Se-Te@Au nanorods and cause cell death; furthermore, the confocal image (Figure 10d) confirms the results. These results were also presented in previously published reports [70,71].

## 4. Conclusions

The formation of well-dispersed and uniform-sized Se-Te and Se-Te@Au nanorods is reported. The Se-Te and Se-Te@Au nanorods exhibited good absorbance in the near-infrared region, and excellent photostability and biocompatibility. After bombarding with NIR light, the temperature of the solution rose up to 60 °C within 10 min, which efficiently destroying bacterial strains and cells reaching almost 99% lethality, in return showing the highest rate of anti-bacterial activities in both Gram-negative *E. coli* and Gram-positive *S. aureus*. The results also showed largest recorded inhibition zones of 25 ± 2 mm and 22 ± 2 mm, respectively, under all conditions, with light, without light, using NIR and without NIR light. The Se-Te@Au rod’s explosion in Hela cells was extensively repressed and demonstrated high toxicity at 100 µg/mL for 5 min when, subjected to an NIR laser. More research is required to confirm our results in in-vivo model studies using Se-Te@Au to develop a new method to solve the existing world challenge of the cancer problem in the medical field. Furthermore, research in the field of mycology and virology must be taken into great consideration to check the stability of antivirals and antifungals against these nanorods.

## Figures and Tables

**Table 1:** A comparison of the anti-bacterial activities in the current study and in previously reported studies.

Strain	Nanoparticle	Zone of Inhibition (Current Study)	Zone of Inhibition (Previously Reported Studies)
*E. coli*	Se-Te	24 ± 2 mm	13 ± 0.5 mm [60]; 9.1 ± 1.6 mm [61], 18 ± 2 mm [62]
	Se-Te@Au	25 ± 2 mm	
*S. aureus*	Se-Te	21 ± 2 mm	10.0 ± 1.2 mm [60]; 9.2 ± 1 mm [61], 14 ± 2 mm [62]

## Data Availability

The data presented in this study are available upon request from the corresponding authors.
